# The Healing Elements of an Eclectic Life Skills Programme: Clients' Perspectives

**DOI:** 10.1155/2024/1499566

**Published:** 2024-08-08

**Authors:** Alta Stone, Lana van Niekerk

**Affiliations:** Division Occupational Therapy Department of Health and Rehabilitation Sciences Faculty of Medicine and Health Sciences Stellenbosch University, P.O. Box 241, Cape Town 8000, South Africa

**Keywords:** group therapy, mental health, occupational therapy, phenomenology, psychosocial rehabilitation, user perspective

## Abstract

**Introduction:** The article reports the healing elements of an eclectic life skills programme (ELSP) from the perspective of group members. An ELSP utilising *open* groups was developed to manage clients with mixed diagnostic profiles and different stages of recovery simultaneously. The aim was to explore the healing elements of an ELSP.

**Methods:** Maximum variation purposive sampling was used to select six participants for the phenomenological inquiry. Data collection is comprised of observations, semistructured interviews, and reflective journals. Data analysis comprised an inductive thematic analysis.

**Consumer Involvement:** Participants all attended groups offered within the ELSP. They participated in two semistructured interviews: the first interview in the week following admission and the second just before discharge. In addition, they documented their experiences in reflective journals for the duration of their participation.

**Findings:** The analogy of a kaleidoscope portrayed the four themes; three pertained to structural dynamics, namely, *programme mirror*, *facilitator mirror*, and *mirror of other group members*. The fourth theme, namely, *the magical pattern*, pertained to personal sense-making by individual group members.

**Conclusions:** The dynamic interplay of healing factors, captured in the themes, facilitated healing. Self-reflection was integral to the creation of a bespoke, facilitated self-learning process with direct application in group members' own lives.

**Summary**

• Service users' perspectives on the value of participation in an eclectic life skills programme.
• Experience-based demonstration of the usefulness of insights developed from the experiences of others.
• Evidence supporting the value of open groups.

## 1. Introduction

Occupational therapy group intervention for mental health service users is well established [1–4] and arguably one of the most frequently used components of occupational therapy intervention in mental health [2, 4]. Life skills groups comprise a wide range of topics using varied group structures [5–8]. In the 1990s, studies yielded positive and quantifiable results for the use of life skills programmes, including anxiety management, stress management, and assertiveness training [9–11]. More recent studies noted positive behaviour changes with the use of community-based life skills training [12] and structured psychoeducational groups [13, 14]. Two reviews [15, 16] on psychoeducation for persons with schizophrenia confirmed better compliance with medication, fewer readmission, shorter hospitalization, an improved mental state, and better functioning. Conversely, limitations in the evidence supporting the effectiveness of group intervention in general have been noted [4, 14, 17, 18]. The study is aimed at contributing research evidence for using life skills groups in occupational therapy.

In South Africa, a growing demand for psychosocial rehabilitation services, coupled with limitations in service availability, necessitates pragmatic approaches in group therapy. One example is an eclectic life skills programme (ELSP) with relatively large open groups (12–15 group members) comprising members with varied diagnoses who are at different stages of recovery. Yalom [19] conceptualised open groups as those that maintain “a consistent size by replacing members as they leave the group” and “there may be a complete turnover of group membership and even of leadership” (p. 277).

Køppe [20] described eclecticism as a “combination of different theoretical starting points” (p. 1). Mikolajewska [21] remarked on the freedom with which an eclectic approach can be used to meet individuals' needs but warned that sound theoretical principles and clinical reasoning are required. The biopsychosocial model [22], with its focus on knowledge, attitude, and skill, together with the volitional subsystem of the model of human occupation [23], contributed to the theoretical foundation of the ELSP. The cognitive behavioural approach (CBT) was predominantly used, and mindfulness principles were applied. Rooted in Beck, Ellis, and Bandura's theories, Cole's description of a CBT approach in dynamic occupational therapy group interventions guided the interpretation of this study. It states that “changes in thinking will produce adaptive changes in client behaviour” ([24], p. 156). During group discussions, the paradigms of the self, the world we live in, and the future were investigated and challenged.

**Figure 1:**
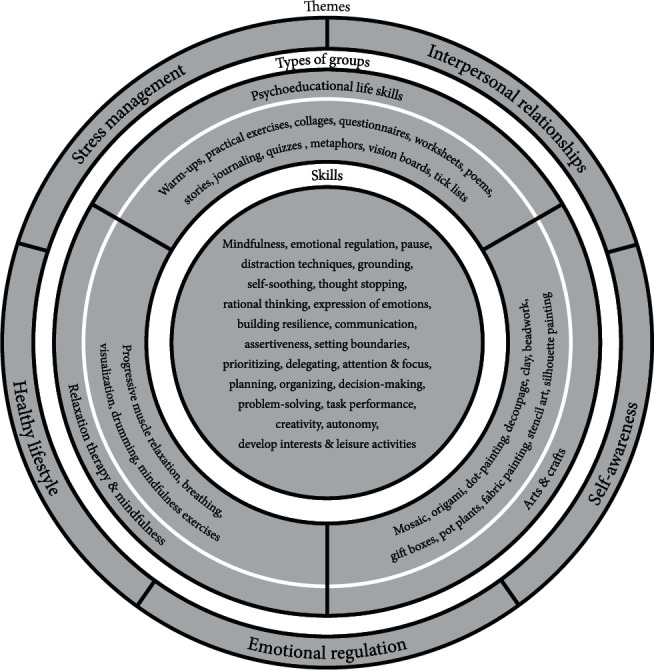
Overview of group therapy programme.

The ELSP, facilitated by two occupational therapists, was structured around five themes, one theme per week: stress management, interpersonal relationships, emotional regulation, self-awareness, and healthy lifestyle. Four groups daily comprised two life skills psychoeducational groups, one craft session, and one relaxation therapy session. The psychoeducational groups fit the description by Burlingame, Kapetanovic, and Ross [25]: “providing information about an illness or problem and teaching coping strategies” (p. 392). Discussion-based groups aimed to develop intellectual and emotional insight through a process of introspection. Homework assignments and personal goals were discussed at the end of each group, and reflective journals were used to monitor the healing process. Craft sessions with creative activities that fit the theme of the week provided a relaxed environment for life skills principles to be practiced in a real-life situation. Daily relaxation therapy sessions provided an opportunity to explore and practice a variety of techniques. Because in-hospital rehabilitation is generally limited in duration, open groups were used in the ELSP to optimise access to life skills groups. An overview of the programme is captured in Figure 1.

Although open groups are used in occupational therapy, little research has been done to explore their use. An Irish survey found that 60% of the groups occupational therapists offered were open groups that allowed clients to select the sessions they wished to join [1]. Members of an open group offered in an acute psychiatric facility experienced the changes in membership of open groups as encouraging; members leaving the group were seen as a sign of progress made [26]. Craft groups that were offered with an *open-door approach* were shown to improve attendance, participation, and autonomy and reduce waiting times [26]. Open groups could thus pragmatically improve access in contexts characterised by limitations in the availability, accessibility, and affordability of rehabilitation.

The research reported here was undertaken to explore the healing elements of the ELSP, a life skills programme using an open-group approach. Egnew's [27] description of healing provided guidelines from which the healing process was understood and explored, namely, “healing is an intensely personal, subjective experience involving a reconciliation of the meaning an individual ascribes to distressing events with his or her perception of wholeness as a person” (p. 255). Furthermore, Jacobson and Greenley's explanation added that “consumers of mental health services who describe themselves as ‘being in recovery' or ‘on a journey of recovery', suggest that the key conditions in this process are hope, healing, empowerment, and connection” ([28], p. 482). The healing elements were captured from the reported experiences of group members who identified elements of the life skills programme they perceived to contribute to their own healing; group facilitators also recorded elements they identified during observation.

## 2. Methods

A qualitative phenomenological study with an interpretivist orientation was undertaken.

### 2.1. Positioning of the Researcher

The first author performed the roles of both group facilitator and researcher, thus making her an “insider” researcher having to mitigate potentially problematic power dynamics. Potential advantages and disadvantages associated with *insider* and *outsider* researcher positions were noted, including that neither of these positions is deemed more, or less, desirable [29]. An obvious risk associated with the insider position is that participants might withhold negative experiences and/or overstate positive experiences. Conversely, benefits of the insider position include already established relationships that promote rapport, which could mean that participants might be more likely to share experiences openly; familiarity with participants providing tacit knowledge, which could deepen understanding; and closeness to the research contexts, which could enhance objectivity, authenticity, and reflexivity [29]. Strategies used to limit the potential negative impact of the insider position are mentioned here and elaborated on in other relevant sections. Informed consent included written and verbal information emphasising the value of participants' actual experiences rather than a mere affirmation of the programme. Time was taken to establish rapport during interviews, and participants were probed to share negative or neutral experiences. Data collection methods and sources were triangulated to include experiences for which the researcher was not the facilitator. Data analysis commenced after the first interview and was done in parallel with data collection; it included peer debriefing by the second author, who was not involved in the ELSP in any way. The second author participated in all stages of the research process.

### 2.2. Research Population

Occupational therapy clients with an Axis 1 diagnosis according to DSM IV R (excluding dementia, psychosis, manic behaviour, and mental retardation) with moderate to mild symptoms who have a score of 51 or higher on the Global Assessment of Functioning (GAF) Scale for Axis V of DSM IV and who participated in the ELSP. The research was conducted in an acute, short-term treatment facility where treatment was funded privately and benefits for in-hospital treatment were limited; the average length of admission period in the clinic was 11–12 days. Most clients in the clinic joined the programme the day after admission.

### 2.3. Sampling

Purposive, maximum variation sampling was used to select six participants. Individuals were selected on their first day of joining the programme for their first-hand experience of the phenomenon. They were invited to participate in the research, and their willingness to share their knowledge, opinion, and insight was discussed before consent forms were signed. To reflect the diversity of the research population, variation was sought in terms of diagnosis, age, sex, and ethnicity.

### 2.4. Data Collection

This comprised interviews, journaling, and observation. Two semistructured interviews (30–45 min) were conducted by the first author, the first during Week 1 following admission and the second just before discharge. The interviews comprised six questions shown in Table 1. Reflective journals followed a structured guideline included in Table 1. Direct observations of participants' functioning, knowledge acquisition, attitude, and skill development were captured by the first author in daily progress notes and summarised in an initial and final report for each participant.

### 2.5. Data Analysis

The first author transcribed the data collected from two interviews, reflective journals, and observations of all six participants. Data collection and analysis occurred concurrently. The inductive thematic analysis comprised six phases [30]. Initial coding and categorisation were done by the first author using Weft QDA, an open-source qualitative analysis software package. Themes and subthemes were refined in consultation with the second author to ensure that the themes cohered meaningfully. No new themes emerged during the analysis of data obtained from Participants 4 to 6, thus suggesting data saturation had been achieved.

### 2.6. Trustworthiness

Trustworthiness criteria comprised credibility, transferability, dependability, and confirmability [31]; these were obtained using triangulation, prolonged engagement, reflexivity, member checking, peer debriefing, providing a rich description of the context of the study, and an audit trail that captured the research process.

Ethical approval with reference number S14/03/057 was obtained from the Health Research Ethics Committee of Stellenbosch University.

## 3. Findings

The demographic details of participants are depicted in Table 2.

In the final phase of data analysis, the use of a kaleidoscope as a metaphor came to mind as it allowed for the synthesis of the four themes in a creative way to demonstrate the dynamic interplay between the healing elements. Three themes comprised healing elements pertaining to the structure of the ELSP, namely, *programme*, *facilitator*, and *other group members*. Using a kaleidoscope as a metaphor, these are depicted as three mirrors.

On the basis of a kaleidoscope, three connected mirrors form triangular planes—when turned, these create ever-changing magical patterns. When looking through a kaleidoscope, there is a captivating moment when it all comes together and you see the picture clearly.

The fourth theme captured participants' internal healing as an individualised process unique to each person.

The wonder of the kaleidoscope lies in the fact that although we may all start with the same parts and components when we turn it, you and I may see different pictures.

In the ELSP, it was the dynamic interaction between themes that created the *magical pattern*. When the clients' specific needs were addressed, they could *see the picture clearly*, which stimulated the process of self-reflection.  Table 3 provides a summary of the four themes and the subthemes they comprise.

The authors prioritized the clients' own voices in this article to describe their subjective experience of the ELSP. Therefore, all quotes present originated from either the interviews or participants' reflective journals. The therapist's observations of the group, as captured in her notes, confirmed their experience.

### 3.1. Theme 1: The Programme Mirror

#### 3.1.1. Big Picture

The ELSP was experienced to foster healing by providing a structured, safe, and stable environment. The holistic approach, including discussion-based groups, arts and crafts, and relaxation therapy, contained elements needed to address participants' needs and provided a balanced day programme.

I see value in the whole programme. It must be regarded as a unit. (Interview Willem)

This is an intensive programme. … I am in a better position; it is because of the holistic programme. (Interview Sipho)

#### 3.1.2. Social Environment

Participants valued the groups, felt they belonged, and experienced support. The open groups included group members in different stages of recovery; this fostered a collective experience of healing and tended to give hope to new members joining.

…not everyone is new. You know those people have been here for a few days. They have already started with the process, and they know what to do. So, it's not that everyone here is now confused and don't know what to do. This actually makes it easier to join. (Interview Willem)

#### 3.1.3. Interesting Topics

Participants identified specific healing properties, which they linked to the group topics and the layout of the programme, including:

• *Important life skills*:

All the topics put together created a very informative programme, especially for such a short time. It is indeed a very good summary of all the life skills. (Interview Willem)

• *Universality: anybody can benefit*:

This is actually a chart – a life chart. (Interview Hassiem)

• *Alleviating doubts about admission*:

My first day here was a real shock to me. I want to get rid of all the anxiety, all the rage in me. But I just don't get the opportunity… And then when I joined the life skills sessions, [the therapist] gave me all the answers. If it wasn't for life skills, I wouldn't have been in such a good space now. … I stayed for the sessions. (Interview Jessica)

• *One theme per week*:

You start with a topic and then finish it. (Interview Willem)

• *Continuity between topics*:

It is concrete on its own, but yet there is a logical flow between [topics]. It tells a story that makes it easy to understand and remember the whole picture. (Interview Willem)

• *Specific goals, but flexible*:

It feels as if we work and then we achieve goals at the end of the sessions. (Interview Jessica)

• *Main frames of reference made explicit*:

I believe that the whole process to change your thought process, is the crux of my recovery process. (Journal Willem)

• *Establishing a theoretical base*:

It stimulates you. Those big words made us feel like we were educated. It is very fancy terms, so when you go out you can also start name-dropping. (Interview Sipho)

It won't help if you explain something in practice to someone, but you don't do it theoretically. You must see and understand. (Interview Johannes)

• *Platform for self-discovery*:

When you go through a difficulty, you always keep something in and you don't talk because you are the solid one at home, you are the wall. And now for the first time you can get to the bottom of it. (Interview Sipho)

I realized that this is a place of inner healing and self-cleaning. (Journal Hassiem)

### 3.2. Theme 2: The Facilitator Mirror

#### 3.2.1. Creating a Therapeutic Milieu

Participants' descriptions of the therapeutic milieu included supportive, casual, nonjudgemental, and friendly; they ascribed the positive group atmosphere to the group facilitator.

I know how children may feel if you ask them a question suddenly and they don't know, and you put them in the “spotlight.” And not once did I feel like, “Oh, dear this could be right, or it could be wrong”. [The facilitator] lets it sound so casual and then everyone is just casual about it. (Interview Jessica)

Participants spoke about the trust and respect that developed between themselves and the group facilitator and explained how the facilitator's presence in the group made them feel safe enough to test and explore skills and approaches that were outside their comfort zones.

[Facilitator] knows how to take you into a new environment or new space without you being too self-conscious. (Interview Sipho)

The facilitator's knowledge of participants' respective psychiatric disorders put them at ease; they picked up three ways in which the facilitator accommodated their conditions, namely, respect and tolerance, structure and guidance, and boundaries.

The structure helps me a lot. I need exact step-by-step… if you skip a step, I've had it. (Interview Hassiem)

[The therapist] don't dig too deep… Because [group members] don't know one another that well personally, it is not fair to expect from us to share confidential information in the group. So, it was just deep enough to benefit from the group. (Interview Jessica)

#### 3.2.2. Choice of Content

Participants appreciated the content of individual group sessions and found the use of an overarching theme to be positive. They recognised the flexibility with which the facilitator directed the programme to meet their needs.

I suspect the facilitator was actually choosing the topic because of exactly me. (Interview Sipho)

[Stress]… that's my problem…, [the topic] fitted me like a glove. (Interview Nellie)

Session topics served as a point of departure for discussions, providing *common ground* for members from diverse language, culture, ethnic, and socioeconomic backgrounds. They appreciated the intellectual stimulation used to introduce life skills principles and found these created a nonthreatening *bridge* between group members.

We are all different sizes and shapes on the outside but very similar on the inside – we all desire the same when it comes to our emotions. (Journal Hassiem)

The emotion I experience is different from what someone else experiences, but the methods to deal with it, remain the same. So, there is common ground. (Interview Willem)

The combination of objective knowledge (research-based facts and theories) and subjective knowledge (personal experience, perspectives, and interpretations) provided a balanced view. The facts and new perspectives helped participants develop new insights, which could be used to improve their own situations.

You may easily just emphasize something or just address a certain point. But if you start with the facts, you are much more inclusive. (Interview Willem)

Participants valued the worksheets used in the groups; these were designed with keywords and symbols that were chosen to trigger curiosity and provide a framework for learning. They stated their preference for short summary pages rather than “wordy” handouts. They remarked on the usefulness of homework assignments. Journaling was experienced as a powerful form of self-expression and a way to monitor progress.

I think worksheets work well,… it forces you to participate… (Interview Willem)

If you only do your homework, you can recover. (Interview Jessica)

After each session I want to put my conclusion in my diary in my own thoughts. … It is going to be my recovery book. So, that book I value highly. (Interview Jessica)

#### 3.2.3. Presentation Style

The facilitator's ability to present information in a creative way and use various teaching styles in the group was appreciated. Inherently complex and abstract concepts were made accessible with the use of simple language, everyday concepts, and practical application.

You get your physical, your visual, auditory, and kinaesthetic learners. [The facilitator] catered for everybody. (Interview Jessica)

So, if you had just told us about it, it would not have stuck in my mind. But when I saw the circles, I made the connection. (Interview Nellie)

Active engagement with information led to the discovery of life principles. Experiential learning was fostered using creative activities and exercises, citing examples participants could relate to and a strong interactive approach.

The icebreaker gets you going so that, when the actual work starts, you are already thinking – your mind is already in the right direction. Then it is very easy to receive the information and you are more relaxed to participate. (Interview Willem)

I immediately understood the story. I will remember that forever because I could relate to the story. (Interview Nellie)

[Active participation] forces you to be honest… it forces you to write something. The encouragement – it forces you to start thinking – to start solving your problems. (Interview Willem)

The therapist's belief in the participants' potential encouraged them to participate, made them feel worthy, and gave them the courage to participate in group-based challenges.

The facilitator conducts the sessions in such a manner that most participants feel very confident that they too, can contribute effectively. (Journal Sipho)

### 3.3. Theme 3: Group Members as Mirror

#### 3.3.1. “All in the Same Boat” Phenomenon

The presence of other people with similar problems fostered healing. The acceptance experienced in the group, regardless of personal background and circumstances, fostered respect and a sense of not being isolated.

…a shared burden is a lighter burden. It's like you know everyone here has a problem. It might not be the same problem, but everyone has a bit of a broken wing. (Interview Willem)

Remember we are taught by society not to look weak. So, outside there you keep a straight face, only to find out when you come here that there are a lot of people with similar stories to you. (Interview Sipho)

#### 3.3.2. “Safety in Numbers” Scenario

The sharing of personal opinions and experiences in the group facilitated a collective healing process. Participants valued that the focus was not on them as individuals all the time; the presence of other group members made them feel safe, and the cohesion provided support.

It becomes a matter of safety in numbers. We are in the same boat, and we are not shy anymore. … And the best thing is when you talk about something, it cures you, it heals you. (Interview Sipho)

You don't get judged here because we all have a problem. So, you're open to say what you want and how you feel. (Interview Johannes)

#### 3.3.3. Creating Synergy

The elements of *give and take* stimulated teamwork and facilitated healing by encouraging participation, supporting each other, and creating opportunities to help others.

I also realized that it's not always about me and my needs only. Others are equally important. (Journal Hassiem)

#### 3.3.4. “Following the Lead” Role Models

Group members at different stages of recovery were considered a visible sign of progress and had a positive effect on participants' moods. Group members who had been in the group for longer tended to share openly and honestly. They were considered role models, and the positive examples they set gave fellow participants hope. Having group members at different stages in the group process was thus considered an advantage. The group norms of tolerance and acceptance encouraged honest sharing.

I think you learn from it because people mention things and you feel exactly the same. When it is one-on-one you just think about yourself, and you have tunnel vision - you might forget things, but when they talk and they name things, it reminds you: “Oh, I have also had that feeling or I have also experienced that”. (Interview Jessica)

### 3.4. Theme 4: The “Magical” Pattern

This theme comprised several categories that were not the same for all group members but converged in different ways to foster individualised healing. Key elements included the following:

• *An individual process*: Although they were together in the same groups, the internal process was personal and individualised.

[The therapist] stirred our brains and stirred our hearts. … And the stirring of my heart may not be the same as for the one next to me. (Interview Jessica)

I feel it is your own process. (Interview Willem)

• *Experiences of autonomy*: Participants valued voluntary attendance. The autonomy to move at their own pace and determine their readiness to participate gave them a sense of control and encouraged them to take responsibility for their own healing.

I said that I am just going to stand and observe, and the therapist said that's OK. And then I thought that I'm just standing there and they are having fun. So, I joined. (Interview Hassiem)

• *Recognising personal needs and challenges*: Experiential learning allowed participants to explore information in a nonthreatening way and helped them to connect with their personal needs.

And my brain went “ping, ping, ping” as I was moved by it. And I thought, “that's me, that's me!” (Interview Jessica)

It feels like everything that is discussed in the class is based on my life. (Interview Johannes)

• *Realization: “I didn't know”*: New knowledge was empowering; it enhanced readiness for change. Group discussions led to the development of new perspectives and fostered the development of new insights that could be used to address problems; such learnings were described as crucial “light bulb” moments that fostered the healing process.

I grew up without any life skills. I had no idea what life skills were until I came to the classes. I mean, I never knew that there were different personality types. I only saw things the way I was brought up. (Interview Hassiem)

I learn something new every day - how to handle situations and the reason why I feel this way. I have never known how to handle certain situations. Now I feel like I know. (Interview Johannes)

• *Experiencing positive change*: The progress that participants experienced motivated them to attend group sessions. Progress was experienced on physical, emotional, and cognitive levels, as well as in the ability to cope with/manage acute symptoms.

Oh, [the relaxation therapy] was so nice. You immediately feel the relief in your body. (Interview Johannes)

I liked the fact that I could make something. That somehow inspired me. (Interview Hassiem)

• *Internalization through application*: Gaining new knowledge provided a concrete starting point for group discussions. However, a crucial step in the process depended on group members applying what they had learnt to their own situations. They had to internalize knowledge to bridge general ideas about life and develop personal beliefs to guide them in everyday life.

I was shocked when I eventually had to apply the diagram to my own life. I thought it through and realized how much I have lost of myself. (Journal Jessica)

It is very interesting information, and it is applicable to you. It's something independent, but you can colour it in as it suits you. You can make it applicable to you – so it will be unique for everyone. (Interview Willem)

• *Responsible for own process and life*: The realization that they were responsible for their own well-being fostered a sense of ownership, which led to a shift in focus from problem-centredness to a solution-based outlook.

I have to be honest. I sometimes duck and dive; hide behind stuff. This place helps you to see that you are in charge of your life and destiny. You make the final decision. You are the pilot of your own life. (Journal Hassiem)

And what is discussed in the class – it's not about being relevant to someone else's life. It affects you directly. So, I want to learn. I am here to pull myself together. (Interview Johannes)

• *Developing skills and self-help tools*: It was the development of coping skills and self-help tools that helped participants connect with their inner strength. Daily practice of skills developed confidence and a sense of competence; it involved the identification of action plans to tackle real-life challenges.

I think the more you start doing things, you feel confident. (Interview Hassiem)

It really gave me the right tools to start helping myself. I already feel “bring it on, world.” I feel, just with the 3 days in the groups, that there are so many relationships that I can restore with the right tools. (Interview Jessica)

## 4. Discussion

Our findings were congruent with other opinions that psychosocial groups are essential for psychosocial rehabilitation [32, 33]. The following central ideas emerged prominently in this study.

### 4.1. The Healing Process

An individualised and personal healing process was fostered within the group context. The programme, the facilitator, and other group members created the structure, but it was the process of introspection that facilitated healing. This was associated with being more hopeful, the development of a positive mindset, an improvement in mood, feeling empowered, and being more confident. Furthermore, the healing was evident in the specific goals and action plans they set to address their individual challenges and needs.

No chronological order or preferred sequence was evident in the elements comprising the internal process of healing. Mental health recovery has been shown not to be linear, and several authors agreed that many pathways might contribute to well-being [34–36]. Clients' own awareness of the healing process, as captured through journaling and verbalised in group sessions and interviews, encouraged them to take ownership and make decisions about their treatment and their lives. They observed this process in themselves and other group members. Monitoring their own progress stimulated a positive attitude and intrinsic motivation to learn and grow. Kelly, Lamont, and Brunero [36] highlighted taking responsibility as an important step in the journey of recovery. Positive experiences while participating in meaningful occupations have been shown to stimulate a sense of control, encouraging clients to take responsibility for feeling better [37, 38].

This study used Kielhofner's [23] understanding of occupation as “doing of work, play or activities of daily living” (p. 5). Clients who participated in the psychoeducation groups often referred to themselves as students of life. Participants expressed a sense of purpose and achievement when they reached goals in the group sessions. The ELSP also provided plenty of opportunities to explore leisure, relaxation, and free-time activities. Positive effects of occupational engagement were documented in several studies [17, 26, 36, 37, 39–41]. Similarly, the positive effect of psychoeducation on emotional regulation, well-being, global functioning, improved mental state, and social functioning has been well described [15, 16, 42, 43].

### 4.2. Content and Layout of the Programme

A “just right” environment facilitating internal healing was created by the facilitator in collaboration with other group members. The programme provided a platform for participants to make sense of their experiences and develop new meaning and direction in their lives. These findings corresponded with several authors' descriptions of key elements of a recovery approach, namely, person-centredness, empowerment, hope, and purpose [35, 44–46].

Congruent with findings by Kelly, Lamont, and Brunero [36], routine was found to contribute to recovery. Participants in the ELSP found that it helped them to organize their *inner worlds* and improve their functioning; they also reported that it provided structure and improved their day–night routines. Our findings also echoed observations by Bryant et al. [17] that occupational therapy groups changed the illness experience by providing something meaningful to do.

The variety of groups (discussion-based, crafts, and relaxation therapy) were deemed all necessary components of the ELSP, and participants valued that the programme was connected and integrated to form one unit. This finding did not feature where only discussion-based groups were studied [5, 13]. However, it was confirmed by Cowls and Hale [14], who suggested that crafts should be added to a psychoeducational programme.

Findings supported in-depth coverage of one theme per week; the order of which was not deemed important as group discussions focused on core principles needed to cope with everyday life, making the information relevant regardless of the specific topic. The programme encouraged critical examination of personal values and assumptions about life, which fostered healing. This is similar to findings by Padilla [47], which indicate that effective learning involves the understanding and critical evaluation of the values and assumptions that underlie knowledge.

Balancing theoretical and practical components of the ELSP fostered learning and supported the finding by Partington et al. [26] that *a place of learning* was a key therapeutic mechanism. Opportunities to practice and master skills to manage stress, regulate emotions, organize day programmes, improve self-awareness and confidence, and improve interpersonal relationships were deemed essential. Participants in the ELSP valued that crafts and relaxation therapy sessions provided a safe environment to try out coping strategies and develop skills. Several studies reported similar positive experiences of relaxation therapy [48, 49]. Arts and crafts lifted participants' mood and functioning as they valued both the creative process and the product. This finding corresponded with a critical review of art-based therapy that similarly captured the benefits in terms of clinical, psychological, social, and occupational recovery [41].

Xia, Zhao, and Jayaram [16] described the purpose of psychoeducation as an opportunity to gain knowledge and understanding through cognitive, affective, and psychomotor processes of learning to create change in attitude, behaviour, and skill. Furthermore, brief psychoeducational interventions were found to improve understanding of mental illness and compliance with treatment [50]. New knowledge fostered intellectual insight; internalization of information led to improved emotional insight. Similarly, psychoeducation was shown to have the potential to develop clients' insight and challenge personal perspectives [13]. Padilla [47] emphasised that the focus should be on what is learnt, not on what is taught.

Findings confirmed the relevance of cognitive behaviour therapy principles as all participants made a direct connection between their beliefs and mental health. Several studies, including some that produced high-quality evidence, supported the effectiveness of CBT for the treatment of psychiatric disorders [51]. Similarly, Ikiugu and Nissen [52] found that cognitive behaviour models were associated with the highest frequency of goal achievement in occupational therapy interventions. Mindfulness principles facilitated self-regulation of symptoms of anxiety and depression and have been reported to be effective in managing depression and anxiety [51, 53].

In line with previous research [38, 39, 42], participants welcomed opportunities provided within the ELSP to practice and develop skills in a safe and supportive environment. Congruent with Spalding, Di Tommaso, and Gustafsson [54], they developed confidence through skill application, which fostered resilience and bolstered their readiness to deal with real-life challenges. Several studies [4, 38, 55] highlighted the need to master self-management skills. Linde [42] emphasised skill development as a goal to equip clients for self-management of mental health when using a CBT frame of reference. Kelly, Lamont, and Brunero [36] confirmed the use of occupational therapy programmes as a platform for reengaging in life.

Our findings confirmed the value-added nature of occupational therapy provided within a group context as the contributions of fellow group members created a sense of belonging and acceptance that fostered a shared process of healing. Partington et al. [26] similarly found that group members found it reassuring when fellow group members shared similar experiences. Linde [42] reported a good fit between Yalom's curative factor of universality and cognitive behavioural principles. Participants in this study agreed that the realization that other group members similarly struggled with mistaken beliefs helped to break down defence mechanisms and stimulate change. However, participants in the ELSP appreciated the tolerance that allowed them to share when they were ready. Cowls and Hale [14] described that it was important to take the phase of illness and participants' readiness to participate into consideration; they advocated for voluntary attendance. Acceptance, support, and a space for self-expression emerged as important therapeutic mechanisms; these were confirmed by the findings of a qualitative research synthesis [38].

Our findings highlighted the pragmatic benefits of using an open-group approach in occupational therapy. Time efficiency was improved by allowing group participation at any time after admission. “Older” group members maintained a stable group environment in which group norms had been established; this encouraged new members with acute symptoms of mental illness to attend. Members at different stages of recovery, participating in the same group, explicated the healing process, thus providing peer modelling and serving as inspiration for their own recovery. Spalding, Di Tommaso, and Gustafsson [54] similarly noted that peer mentoring, whether through discussing progress or observing others, encouraged group members to participate.

### 4.3. Style of Presentation

Participants appreciated the interactive approach with variations in auditory, visual, and kinetic teaching styles that kept them engaged; they also valued the creative ways used to explain abstract and complex concepts. Morris [56] also combined art-based and CBT techniques, noting that it provided verbal and nonverbal expression. Similar to Cowls and Hale [14], our findings showed that activities, exercises, and examples produced memories that facilitated internalization and retention of information. Worksheets improved the retention of key concepts; this finding was shared by Cowls and Hale [14], who showed that sufficient repetition and written handouts improved the absorption of information. The benefits of incorporating homework tasks into a CBT approach are well documented [51, 57]. Kelly, Lamont, and Brunero [36] noted that these enhanced the journey of recovery in a community-based therapy setting.

Our findings showed that journaling, in which words, symbols, and visual illustrations were combined, helped participants capture insights and monitor progress. Similarly, Deaver and McAuliffe [58] found journaling that combined imagery and writing provided a means for self-expression and connection with “that which cannot be said in words” (p. 627). Chirema [59] also found journaling useful for reflection and learning.

Participants appreciated the facilitator's skilled approach; although they were challenged, they felt accepted as they were allowed to participate in accordance with what they could tolerate. They noted that the facilitator's respect for their stage in the recovery process maintained their self-respect. This was in line with recovery-oriented practice, in which the therapist takes positive risks while maintaining the consumer's right to choose, as described by Nugent, Hancock, and Honey [45]. Spalding, Di Tommaso, and Gustafsson [54] also reported that clients valued being part of the therapy decision-making process. Conversely, Kelly, Lamont, and Brunero [36] argued that it was necessary to challenge clients gently to stimulate a process of recovery. Wimpenny, Savin-Baden, and Cook [38] emphasised the importance of clinical reasoning in determining when a challenge should be therapist- or user-led.

### 4.4. Methodological Considerations—Limitations

Despite strategies to mitigate the potential impact of the first author's positioning as a group facilitator–researcher, the sharing of negative experiences might have been suppressed.

Although the relatively small sample size could limit the credibility of findings, persistent observation, triangulation, and member checking were used to improve the trustworthiness of findings.

The variability of programme content and facilitator strategies limits the confirmability and transferability of our findings.

The occupational therapy facilitators of the ELSP were skilled and experienced, contributing directly to positive experiences of healing.

## 5. Conclusion

The flexibility of the ELSP programme allowed for the pragmatic accommodation of diverse client needs in a fast-paced, acute mental health environment. This improved access and optimal use of rehabilitation resources. The combination of a cognitive behaviour approach and mindfulness principles optimized participation in an environment with a regular change in group membership brought by daily admissions and discharges. The ELSP optimized short-stay admissions by teaching coping strategies. Homework assignments and journaling promoted the development of personal insight and retention.

## Figures and Tables

**Table 1:** Questions that guided data collection.

Questions used during semistructured interviews:
1. Describe your experience on the first day in the life skills programme. 2. What made you come back to the group the next day? 3. What is your opinion about the topic and the use of activities, worksheets, and homework assignments in the life skills programme? 4. What role, if any, does the life skills programme play in your recovery process? 5. Were there any specific benefits or healing elements that you experienced when therapy was conducted within a group setting? 6. What were the most and least valuable aspects of the life skills programme?

Guidance provided for journaling:
Please capture your “lived” experience in your own words; you may focus on the topic, activities and worksheets, experience in the group, your own recovery process, personal growth and development, and considerations when contemplating joining the group tomorrow.

**Table 2:** Demographic details of participants.

**Pseudonym/sex/age/occupation**	**Axis 1 diagnoses**	**Days attended**
Willem/male/21/university student	Major depressive disorder	9
Jessica/female/36/teacher	Major depressive disorder and somatoform disorder	7
Hassiem/male /49/technician	Obsessive–compulsive disorder and attention deficit disorder	5
Sipho/male/49/qualified journalist working as a clerk in human resources	Major depressive disorder and posttraumatic stress disorder	15
Nellie/female/50/recent early retiree	Major depressive disorder and Panic disorder	10
Johannes/male/24/mine worker (working underground)	Major depressive disorder, generalized anxiety disorder, and alcohol abuse	6

**Table 3:** Themes and subthemes.

**Theme 1: The programme mirror**	**Theme 2: The facilitator mirror**	**Theme 3: Other group members mirror**	**Theme 4: The “magical” pattern**
Big picture	Creating a therapeutic milieu	“All in the same boat” phenomenon	An individual process
Social environment	Choice of content	“Safety in numbers” scenario	Experiences of autonomy
Interesting topics	Style of presentation	Creating synergy	Recognising personal needs and challenges
		“Following the lead” role models	Realization: “I did not know”
			Experiencing positive change
			Internalization through application
			Responsible for own process and life
			Developing skills and self-help tools

## Data Availability

The data that support the findings of this study are not publicly available due to privacy restrictions.
